# Medullary Serotonergic Binding Deficits and Hippocampal Abnormalities in Sudden Infant Death Syndrome: One or Two Entities?

**DOI:** 10.3389/fped.2021.762017

**Published:** 2021-12-21

**Authors:** Robin L. Haynes, Hannah C. Kinney, Elisabeth A. Haas, Jhodie R. Duncan, Molly Riehs, Felicia Trachtenberg, Dawna D. Armstrong, Sanda Alexandrescu, Jane B. Cryan, Marco M. Hefti, Henry F. Krous, Richard D. Goldstein, Lynn A. Sleeper

**Affiliations:** ^1^Department of Pathology, Boston Children's Hospital and Harvard Medical School, Boston, MA, United States; ^2^Department of Research, Rady's Children's Hospital, San Diego, CA, United States; ^3^Latrobe Regional Hospital, Traralgon, VIC, Australia; ^4^HealthCore, Inc., Watertown, MA, United States; ^5^Department of Pathology (Emeritus), Baylor College of Medicine, Houston, TX, United States; ^6^Department of Neuropathology, Children's Health Ireland and Beaumont Hospitals, Dublin, Ireland; ^7^Department of Pathology, University of Iowa, Iowa City, IA, United States; ^8^Department of Pathology (Emeritus), Rady Children's Hospital, San Diego, CA, United States; ^9^Department of Pediatrics (Emeritus), University of California, San Diego, San Diego, CA, United States; ^10^Department of Pediatrics, Harvard Medical School, Boston, MA, United States; ^11^Robert's Program on Sudden Unexpected Death in Pediatrics, Division of General Pediatrics, Department of Pediatrics, Boston Children's Hospital, Boston, MA, United States; ^12^Department of Cardiology, Boston Children's Hospital, Boston, MA, United States

**Keywords:** medulla, temporal lobe epilepsy, seizure, arousal, dentate gyrus

## Abstract

Sudden infant death syndrome (SIDS) is understood as a syndrome that presents with the common phenotype of sudden death but involves heterogenous biological causes. Many pathological findings have been consistently reported in SIDS, notably in areas of the brain known to play a role in autonomic control and arousal. Our laboratory has reported abnormalities in SIDS cases in medullary serotonin (5-HT) receptor _1A_ and within the dentate gyrus of the hippocampus. Unknown, however, is whether the medullary and hippocampal abnormalities coexist in the same SIDS cases, supporting a biological relationship of one abnormality with the other. In this study, we begin with an analysis of medullary 5-HT_1A_ binding, as determined by receptor ligand autoradiography, in a combined cohort of published and unpublished SIDS (*n* = 86) and control (*n* = 22) cases. We report 5-HT_1A_ binding abnormalities consistent with previously reported data, including lower age-adjusted mean binding in SIDS and age vs. diagnosis interactions. Utilizing this combined cohort of cases, we identified 41 SIDS cases with overlapping medullary 5-HT_1A_ binding data and hippocampal assessment and statistically addressed the relationship between abnormalities at each site. Within this SIDS analytic cohort, we defined abnormal (low) medullary 5-HT_1A_ binding as within the lowest quartile of binding adjusted for age and we examined three specific hippocampal findings previously identified as significantly more prevalent in SIDS compared to controls (granular cell bilamination, clusters of immature cells in the subgranular layer, and single ectopic cells in the molecular layer of the dentate gyrus). Our data did not find a strong statistical relationship between low medullary 5-HT_1A_ binding and the presence of any of the hippocampal abnormalities examined. It did, however, identify a subset of SIDS (~25%) with both low medullary 5-HT_1A_ binding and hippocampal abnormalities. The subset of SIDS cases with both low medullary 5-HT_1A_ binding and single ectopic cells in the molecular layer was associated with prenatal smoking (*p* = 0.02), suggesting a role for the exposure in development of the two abnormalities. Overall, our data present novel information on the relationship between neuropathogical abnormalities in SIDS and support the heterogenous nature and overall complexity of SIDS pathogenesis.

## Introduction

The sudden and unexpected death of an apparently healthy infant during a sleep period has long been recognized as a medical entity requiring investigation, but its cause remains unknown. Since the middle of the twentieth century, various definitions have been proposed for this phenomenon. It has usually been labeled as a “syndrome,” which is a set of medical signs and symptoms that correlate strongly with each other without an established unifying cause. The use of the word syndrome is distinct from “disease,” which is utilized when the cause or mechanism of the signs and symptoms is known, either by diagnostic laboratory findings, or pathognomonic clinical and/or autopsy findings. The typical phenotype of sudden infant death syndrome (SIDS) is the unique age distribution with a peak at 2–4 postnatal months, occurrence of death associated with a sleep period, socioeconomic disadvantage, and male predominance. SIDS is a diagnosis of exclusion and its differential diagnosis is broad and heterogeneous, including various causes that may be found on autopsy, e.g., inborn errors of metabolism, congenital heart disease.

**Figure 1:**
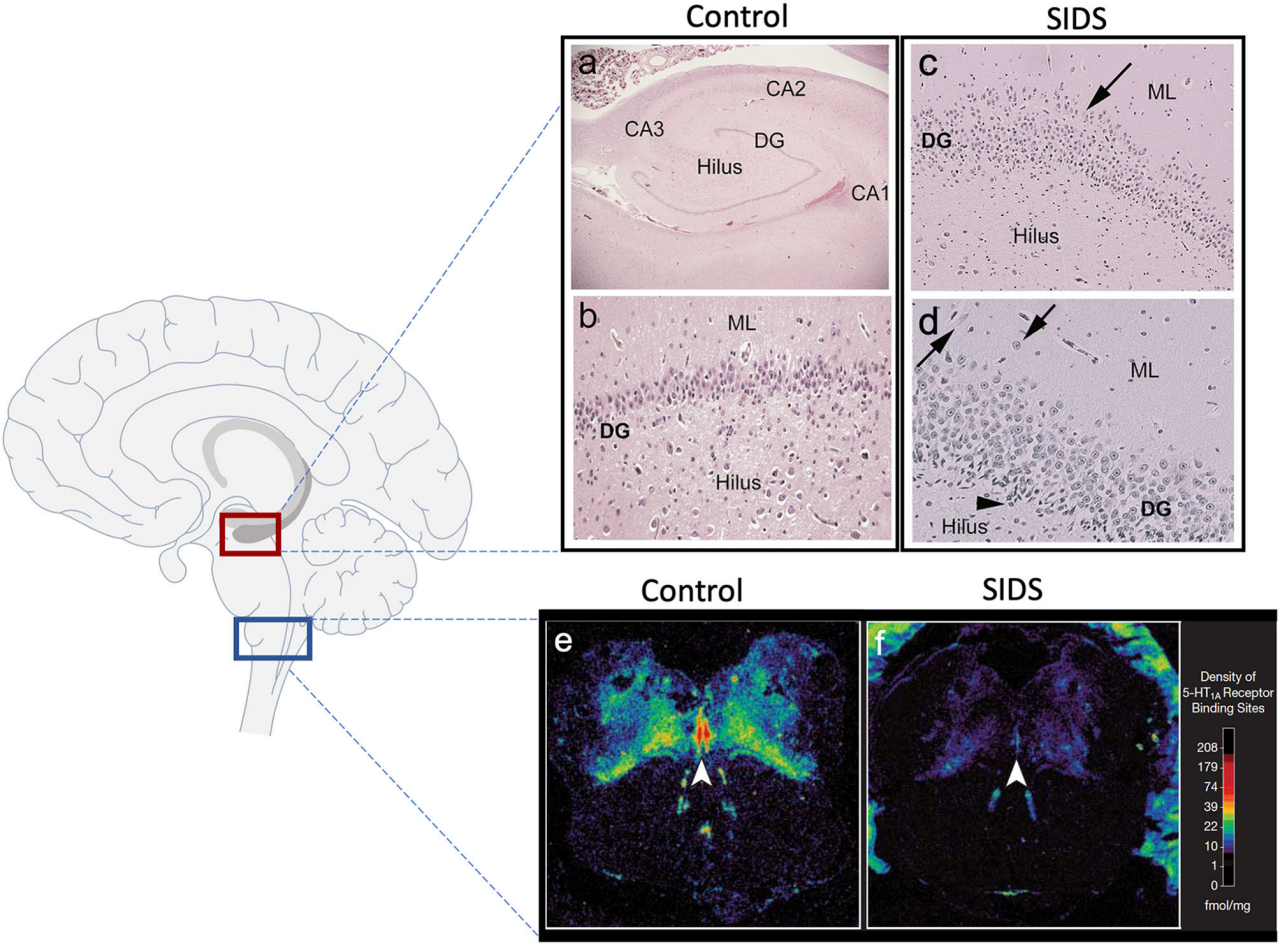
Hippocampal and medullary pathology identified in SIDS infants. The diagram on the left shows general brain structure with the hippocampus and medulla highlighted with red and blue boxes, respectively. Images **(a–d)** show hematoxylin and eosin staining of the hippocampus. Hippocampal anatomy is shown at 4x in a control infant for reference **(a)**. The dentate gyrus (DG) in a control infant is shown at 10x with a densely packed, single layer of granule cells **(b)**. The hippocampus of a SIDS infant displays **(c)** bilamination of the DG (arrow), **(d)** single, ectopic granule cells in the molecular layer (arrow), and **(d)** progenitor cells in the subgranular layer (arrowhead). Hippocampal images were modified from Kinney et al. (8). Examples of medullary 5-HT_1A_ autoradiography are shown in **(e,f)**. The density of 5-HT_1A_ receptor binding, including in the raphe obscurus (arrowhead), is visually lower in a SIDS case **(f)** compared with a control **(e)**. The receptor binding surrounding the control medulla **(e)** represents binding in cerebellum tissue. A colored density scale is included for reference. Brainstem images were modified with permission from Paterson et al. (4). DG, dentate gyrus; CA, cornu ammonis; ML, molecular layer.

Over the last two decades, our group has provided substantial evidence using neurochemical techniques that a subset of SIDS infants is characterized by serotonergic brainstem pathology in regions of the medulla oblongata involved in cardiorespiratory control and arousal. These abnormalities include serotonin (5-HT) receptor binding abnormalities (1–5), a decrease in 5-HT levels and tryptophan hydroxylase 2 (TPH2) (5), the key regulatory enzyme in 5-HT production, and an increase in serotonergic cells with an immature-like phenotype (4). Among these, the most robust and reproducible serotonergic abnormality identified in the brainstem to date is a deficiency in binding to the 5-HT_1A_ receptor (4, 5), a receptor which functions as a presynaptic auto-receptor on 5-HT neurons and a heteroreceptor on postsynaptic 5-HT neurons and non-5-HT neurons (6). This binding deficiency has been identified by us with tissue receptor autoradiography in two independent published datasets of SIDS cases compared to non-SIDS controls (4, 5) and confirmed by other laboratories with different techniques (7). Most recently, our laboratory reported a novel anatomic finding from light microscope studies in the hippocampus of ~40% of SIDS cases (8). Hippocampal abnormalities, including abnormalities of the dentate gyrus (DG), have been reported in other cohorts of SIDS and sudden unexpected death in childhood (SUDC) (9–15), suggesting hippocampal involvement across a spectrum of ages. The hippocampal abnormalities identified in SIDS and SUDC, specifically granular cell bilamination of the DG, had been reported in patients with temporal lobe epilepsy (TLE) (16–19), suggesting a seizure-related mechanism of sudden death in SIDS and SUDC, a hypothesis postulated by others (20, 21). Shown in Figure 1 are examples of medullary 5-HT_1A_ binding and the hippocampal features analyzed here. While hippocampal pathology in SIDS suggests an involvement of sleep-related fatal seizures, brainstem serotonergic abnormalities suggest brainstem-mediated central cardiorespiratory dysfunction during sleep.

Biologically, hippocampal development and the brainstem serotonergic system are related through the trophic actions of 5-HT during development (22–25). Pathologically, they are related through 5-HT-mediated cardiorespiratory dysfunction during seizures and seizure-induced impairment in serotonergic brainstem function (21, 26). Functionally, medullary 5-HT and limbic sites including the hippocampus are considered to be interconnected “nodes” and comprise an integrated central homeostatic network that regulates responses to stress (27). Given the links between the hippocampus, brainstem 5-HT and serotonergic dysfunction during seizures, we postulated that the hippocampal abnormalities identified in a subset of SIDS infants are related to the brainstem serotonergic abnormalities identified in SIDS infants. This is based on the fact that SIDS infants with hippocampal abnormalities seem to share clinical presentations (sudden and unexpected death), demographics, and general autopsy findings with SIDS infants with medullary 5-HT_1A_ abnormalities. While these common features suggest one pathological process, whether they represent two separate diseases is unknown. Whether hippocampal abnormalities coexist with brainstem serotonergic abnormalities in the same infant is also unknown. In this study we hypothesized that the medullary 5-HT_1A_ binding abnormality is found in SIDS infants with hippocampal structural abnormalities, suggesting a dependence between the two lesions and providing evidence for a single entity with a combined hippocampal-brainstem phenotype.

Prior to our analysis of the relationship between medullary and hippocampal abnormalities in SIDS, we expanded upon our reported medullary 5-HT_1A_ findings to show 5-HT_1A_ binding deficiencies in a combined published and unpublished cohort of SIDS and controls. Subsequently, in order to investigate the hypothesized relationship between the hippocampal findings and brainstem 5-HT_1A_ abnormalities, this research investigated three specific questions: (1) do SIDS cases with identified hippocampal abnormalities have lower medullary 5-HT_1A_ binding compared with SIDS cases without abnormalities?; (2) are SIDS cases with the lowest medullary 5-HT_1A_ binding in the SIDS cohort at higher risk for hippocampal abnormalities compared with SIDS cases with normal or elevated binding?; and (3) are there clinical and/or risk factors specifically associated with the concurrent presence of both abnormalities? To address these questions, we used an analytic cohort with both a histological assessment of fixed hippocampus (8) and neurochemical analysis of frozen medulla (4, 5). In our analysis of the 5-HT_1A_-hippocampal relationship, we focused specifically on hippocampal features shown to have a higher prevalence in SIDS infants compared with controls, namely, focal granule cell bilamination of the DG, clusters of immature cells in the subgranular layer of the DG, and single ectopic granule cells in the molecular layer of the DG (8). We focused on eight brainstem nuclei reported to be abnormal in SIDS (as determined by 5-HT_1A_ receptor binding), including nuclei containing 5-HT cells and considered by our group as part of the core medullary serotonergic lesion in SIDS (28).

## Materials and Methods

### Tissue

Tissue samples were obtained from infant autopsies between 1998 and 2013. Tissue came from the San Diego Medical Examiner's office (SDME) and were available for research under the auspice of the California Code, Section 27491.41. Deaths adjudicated as SIDS were those in which a complete autopsy, death scene investigation, and review of the clinical history and circumstances of death, failed to reveal a known cause of death (COD) (4, 5). All cases were internally adjudicated using standard protocols, autopsy, and death scene investigations. Adjudications were done blinded to findings in the medulla and the hippocampus.

### Combined Cohort of SIDS and Controls for Analysis of Medullary 5-HT_1A_ Binding

**Figure 2:**
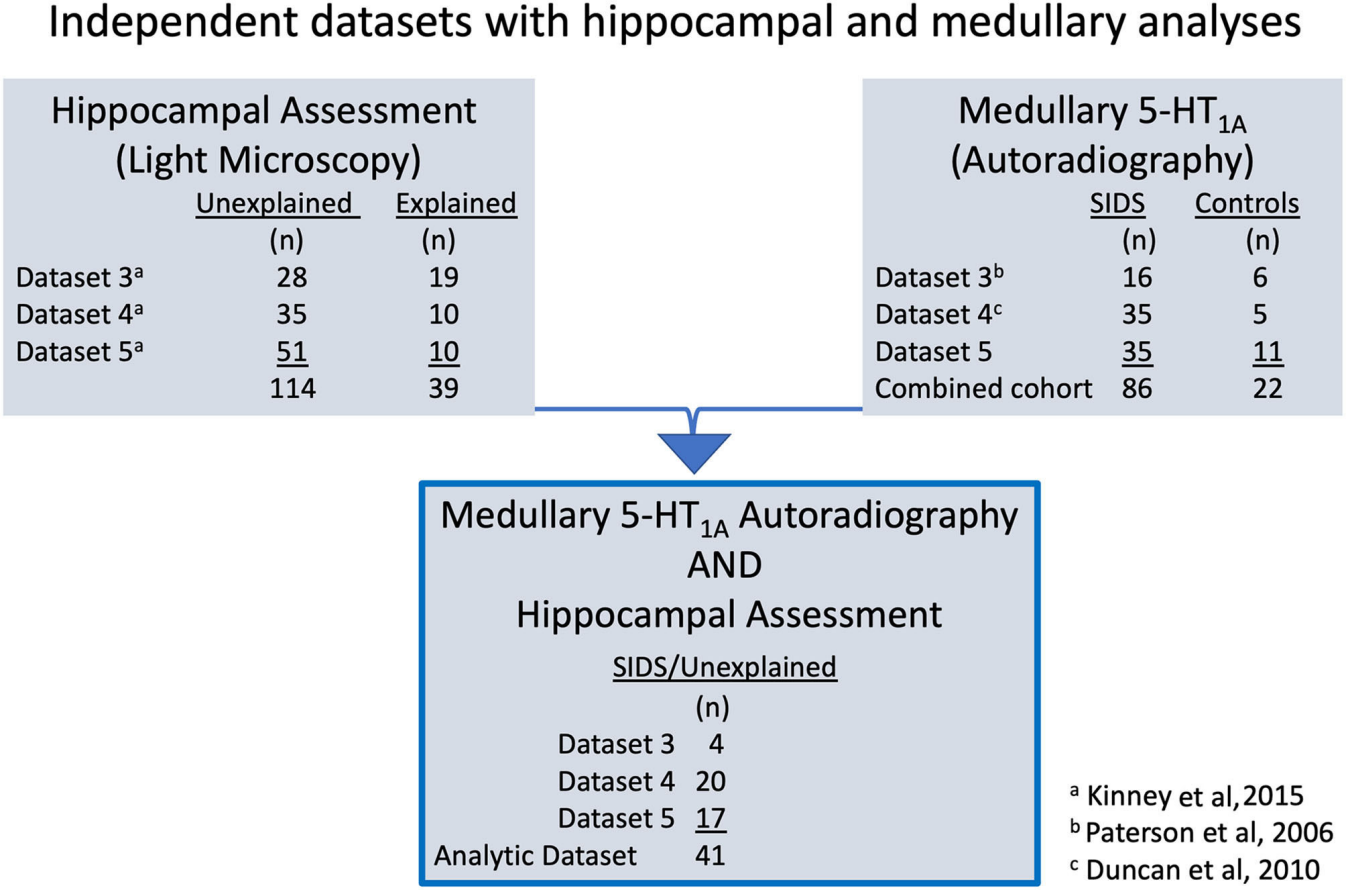
The diagram illustrates the different laboratory datasets from which the analytic cohort (*n* = 41) originates. Datasets 3–5 are independent datasets with no overlap in SIDS or control cases. Fixed hippocampus tissue was available for morphological assessment on a total of 153 cases. Hippocampal results were reported in Kinney et al. (8) using adjudications of unexplained (*n* = 114) and explained (*n* = 39). Frozen medulla tissue was available for 5-HT_1A_ receptor autoradiography on 108 total cases. Results from analysis of medullary 5-HT_1A_ binding data from Datasets 3 and 4 have been previously published in Paterson et al. (4) and Duncan et al. (5), respectively. Additional 5-HT_1A_ binding data from a third independent dataset, Dataset 5, have been collected and added to published Datasets 3 and 4 for the combined cohort of 108, including 86 SIDS cases and 22 controls in this report (See Figure 3). Individual SIDS cases that had available hippocampal assessment data and medullary 5-HT_1A_ receptor binding data comprise the analytic cohort (*n* = 41 SIDS). Of note, all 41 SIDS cases were adjudicated as unexplained in Kinney et al. (8).

Data on 5-HT_1A_ binding levels were obtained from a combined cohort of SIDS (*n* = 86) and control (*n* = 22) cases that originated from multiple, independent datasets collected over different periods of time. The individual datasets comprising the cohort were designated in our laboratory as Dataset 3 [*n* = 22; 6 controls,16 SIDS], including cases collected from 1998 to 2004 (4), Dataset 4 [*n* = 40; 5 controls, 35 SIDS], including cases collected from 2004 to 2008 (5), and Dataset 5 [*n* = 46; 11 controls, 35 SIDS], including cases collected from 2008 to 2013 [unpublished]. Medullary 5-HT_1A_ binding was performed on medulla taken at autopsy then fresh-frozen. Controls in our combined cohort for 5-HT_1A_ analysis were infants that died from a definitive COD. The CODs are as follows: congenital heart disease (*n* = 7); respiratory infection (*n* = 4); asphyxial accident (e.g., wedging of head) (*n* = 4); drowning (*n* = 1); gastroesophageal reflux disease (*n* = 1); complication of prematurity (*n* = 1); fatty acid oxidation disorder (*n* = 1); hemolytic anemia associated with febrile illness (*n* = 1); meconium aspiration (*n* = 1); complications of traumatic placental abruption (*n* = 1). The combined cohort represents all SIDS and control cases to date that originated from the SDME and were analyzed in our laboratory for medullary 5-HT_1A_ binding. Of the 86 SIDS cases in this combined cohort, 41 had hippocampal data available for analysis, as described below in “*SIDS subset with both medullary 5-HT*_1*A*_ *binding and hippocampal analyses”* and depicted in Figure 2.

### Original Dataset of SIDS and Controls for Hippocampal Features

Hippocampal features were originally examined in 153 cases. These cases included 114 cases adjudicated as unexplained deaths and 39 cases adjudicated as explained deaths (8). Histological assessment was performed on formalin-fixed tissue taken at autopsy. The definition of unexplained, as published by Kinney et. al. (8), is equivalent to the definition of SIDS as reported in previous 5-HT_1A_ binding studies (4, 5). In our current study, we use the term SIDS, rather than unexplained, to be consistent with previous published brainstem neurochemistry studies (4, 5). Of the 114 SIDS cases with hippocampal assessment, 41 had medullary 5-HT_1A_ binding measurements available for analysis, as described below in “*SIDS subset with both medullary 5-HT*_1*A*_ *binding and hippocampal analyses”* and depicted in Figure 2.

### SIDS Analytic Cohort With Both Medullary 5-HT_1A_ Binding and Hippocampal Analyses

Forty one SIDS cases had both hippocampal assessment and medullary 5-HT_1A_ binding data and comprise the analytic cohort (Figure 2). This combined SIDS cohort with data from frozen medulla and fixed hippocampus includes the following: Dataset 3 SIDS (*n* = 4) (4), Dataset 4 SIDS (*n* = 20) (5), and Dataset 5 SIDS [unpublished] (*n* = 17).

### Hippocampal Study Review

Hippocampi analyses and data were previously published (8). Briefly, coronal hippocampal sections (6μm) were independently analyzed by pediatric neuropathologists (Kinney, Armstrong). The presence or absence of 44 developmental and acquired features in the DG, Ammon's horn, subiculum, entorhinal cortex, temporal cortex and white matter was assessed for each case (8).

### Brainstem Receptor Autoradiography

Receptor autoradiography for medullary 5-HT_1A_ binding was previously performed on frozen medulla of Datasets 3 and 4 using ^3^H 8-hydroxy-2-[di-N-propylamino]-tetralin (^3^H-DPAT) as described (4, 5). Frozen medulla from Dataset 5 were analyzed using these same protocols. For this report, we focused our analysis on eight nuclei that contain 5-HT-producing neurons (raphe obscurus [RO], gigantocellularis [GC], paragigantocellularis lateralis [PGCL], intermediate reticular formation [IRZ], and arcuate nucleus [ARC]) and nuclei that contain 5-HT projections (nucleus of the solitary tract [NTS], hypoglossal nucleus [HG], and dorsal motor nucleus of the vagus [DMX]).

### Statistical Analyses

#### Medullary Abnormalities in 5-HT_1A_ Across the Combined Cohort of Cases With 5-HT_1A_ Binding

Analysis of covariance was performed to examine differences in mean 5-HT_1A_ binding in SIDS vs. Controls, adjusted for postconceptional age and dataset, as these two variables are potential confounders due to their association with both 5HT_1A_ binding and diagnosis (SIDS vs. Control).

A test of interaction between diagnosis (SIDS vs. Control) and postconceptional age was also performed. Least-squares (adjusted) means with standard error for SIDS vs. Controls were reported for the models involving nuclei that had no age by diagnosis interaction. Slope estimates of 5-HT_1A_ binding as a function of age were reported for the models from nuclei that displayed a significant age by diagnosis interaction.

#### Comparison of Mean 5-HT_1A_ Binding With the Presence or Absence of Specific Hippocampal Abnormalities

To look for an association between low 5-HT_1A_ binding and hippocampal abnormalities, we chose to focus on hippocampal features that were significantly more common in SIDS cases compared with controls (8). These features, thought to be developmental in nature as opposed to acquired (e.g, due to hypoxia), include focal granule cell bilamination, clusters of immature cells in the subgranular layer, and single ectopic granule cells in the molecular layer of the DG. The primary outcomes were 5-HT_1A_ binding values in fmol/mg (continuous outcome) in each nucleus. Multivariable linear regression was used to compare mean 5-HT_1A_ binding in SIDS cases with and without the hippocampal feature, adjusted for dataset and postconceptional age (PCA) [gestational age + postnatal age] (**Table 5**).

#### Association Between the Presence of a Hippocampal Abnormality and Low 5-HT_1A_ Binding

For each nucleus, multivariable logistic regression was used to estimate the association between the binary, 5-HT_1A_ (lowest quartile [Q1] vs. above first quartile) outcome variable and the presence vs. absence of a hippocampal feature, adjusted for PCA. Classification into the lowest quartile was based on the distribution of binding specific to each dataset. Classification of 5-HT_1A_ binding within individual datasets was necessary because of small, but significant, differences in binding data across datasets collected over the 14 year period of case collection and analysis. For **Table 6**, the presence vs. absence of a hippocampal abnormality was instead modeled as the outcome, and for each model the PCA-adjusted predicted probability of having a hippocampal abnormality was reported.

#### Analyses of SIDS Subsets as Defined by the Presence or Absence of Low Medullary 5-HT_1A_ Binding and Hippocampal Features

SIDS cases were grouped into four subsets according to the presence vs. absence of low medullary 5-HT_1A_ binding and presence vs. absence of a specific hippocampal feature. A SIDS case was defined as having low medullary 5-HT_1A_ binding if 5-HT_1A_ binding was in the lowest quartile (Q1) for two or more of the eight medullary nuclei. A test of association between clinical features and SIDS subsets (four groups: presence vs. absence of hippocampal abnormality X binding in Q1 vs. binding above Q1) was performed using a Fisher exact test for categorical features, and a Wilcoxon rank sum test for continuous features.

In all analyses, a *p* < 0.05 was considered statistically significant. Comparisons were not adjusted for multiplicity associated with examination of differences in multiple brain nuclei.

Analyses were performed with SAS version 9.4 (SAS Institute, Inc., Cary, NC) and R version 4.0.3.

## Results

### Medullary Abnormalities in 5-HT_1A_ Across the Combined Cohort of SIDS and Control Cases With 5-HT_1A_ Receptor Binding Data

**Figure 3:**
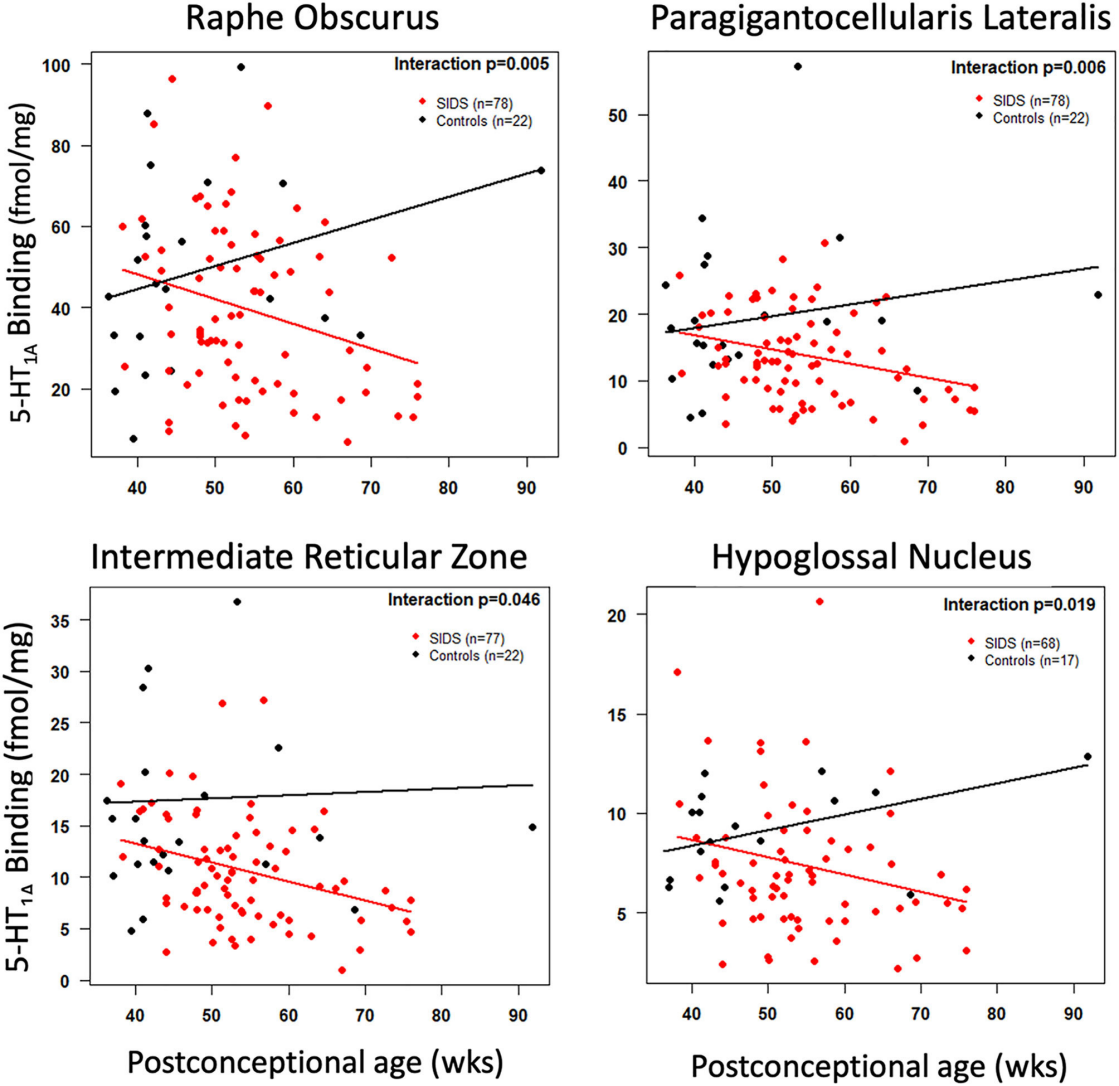
Cross-sectional association of postconceptional age and medullary 5-HT_1A_ binding in selected nuclei. The red and black lines represent the estimated dataset-adjusted mean of 5-HT_1A_ binding according to postconceptional age for SIDS and controls from combined datasets, respectively. The *p*-value reflects a postconceptional age × Diagnosis interaction.

Before analyzing the analytic cohort of SIDS cases with both hippocampal assessment and medullary 5-HT_1A_ binding data, we examined the full combined 5-HT_1A_ cohort of SIDS cases (*n* = 86) for abnormalities in 5-HT_1A_ binding compared to control cases (*n* = 22). The demographics of SIDS and control cases are noted in Table 1. The two groups differed with respect to median PCA; therefore, SIDS vs. control comparisons were adjusted for PCA. Mean postmortem interval (PMI) was higher (*p* = 0.025) in the SIDS cases. As previously published (4, 5), however, there was no effect of PMI on 5-HT_1A_ binding. In two nuclei, the GC and NTS, there was lower PCA-adjusted mean binding in SIDS infants compared to controls (*p* = 0.006 and 0.02, respectively) (Table 2). Statistical analyses of this combined cohort showed a significant age vs. diagnosis interaction in the following nuclei: RO (*p* = 0.005), PGCL (*p* = 0.006), IRZ (*p* = 0.046), HG (*p* = 0.02) (Table 2; Figure 3). Within these nuclei the age vs. diagnosis interaction shows that 5-HT_1A_ binding decreases with PCA in SIDS cases, but binding does not vary with age in control cases. In the remaining two nuclei, the ARC and DMX, there was no interaction and no difference in mean binding between SIDS infants and controls (Table 2).

**Table 1:** Demographics of the SIDS and control cases comprising the full combined cohort for 5-HT_1A_ binding analysis.

	**Controls**	**SIDS**	***p*-value**
	**Mean** **±** **SD or**		
	***n*** **(%)**		
*N*	22	86	
Gestational age (wk)	38.8 ± 1.9	38.3 ± 3.2	0.46
Postnatal age (wk)	9.1 ± 12.8	15.7 ± 8.9	0.006
Postconceptional age (wk)	47.9 ± 13.3	54.0 ± 8.8	0.01
Median (IQR) Postconceptional age (wk)	41.9 (40.3, 53.3)	52.6 (48.0, 58.2)	0.001
Prematurity (GA <37 weeks)	3 (14%)	17 (20%)	0.76
Postmortem interval (hr)	15.5 ± 6.7	19.2 ± 7.0	0.03
Male sex	8 (36%)	14 (64%)	0.15
Race/ethnicity			0.04
White	6 (30%)	36 (44%)	
Black	5 (25%)	9 (11%)	
Hispanic	9 (45%)	23 (28%)	
Other	0 (0%)	13 (16%)	
Unknown	7	5	

*SIDS, sudden infant death syndrome; 5-HT, serotonin; SD, standard deviation; wk, week; PCA, postconceptional age; GA, gestational age; hr, hours. SD, Standard Deviation; IQR, interquartile range*.

**Table 2:** 5-HT_1A_ receptor binding in the medullary serotonin system in a combined cohort of SIDS and controls.

		**Age- and dataset-adjusted mean** **±** **SE**				**Estimated slope** **±** **SE**,	
		**fmol/mg tissue**				**fmol/mg tissue**	
**Nucleus**	**N SIDS/Controls**	**SIDS**	**Controls**	***p*-value**	**Age x Diagnosis Interaction *p*-value^a^**	**SIDS Change in binding per week**	**Controls Change in binding per week**
RO	78/22	–	–	–	0.005	−0.61 ± 0.26^b^	+0.57 ± 0.33
GC	78/22	16.30 ± 1.01	22.36 ± 1.87	0.006	n.s.		
PGCL	78/22	–	–	–	0.006	−0.21 ± 0.09 ^b^	+0.18 ± 0.11
IRZ	77/22	–	–	–	0.05	−0.18 ± 0.07 ^b^	+0.03 ± 0.08
ARC	56/16	5.32 ± 0.40	6.54 ± 0.71	0.14	n.s.		
HG	68/17	–	–	–	0.02	−0.09 ± 0.04 ^b^	+0.08 ± 0.05
DMX	53/11	8.26 ± 0.52	9.55 ± 1.16	0.32	n.s.		
NTS	68/17	10.26 ± 0.54	13.08 ± 1.06	0.02	n.s.		

*All estimates are adjusted for postconceptional age and dataset. Abbreviations. SIDS, sudden infant death syndrome; 5-HT, serotonin; RO, raphe obscurus; GC, gigantocellularis; PGCL, paragigantocellularis lateralis; IRZ, intermediate reticular zone; ARC, arcuate nucleus; HG, hypoglossal nucleus; DMX, dorsal motor nucleus of the vagus; NTS, nucleus of the solitary tract; SE, standard error; n.s., not significant. The numbers (N) of SIDS and controls are given in the second column. The total N varies due to the fact that not every case had binding data available for every nucleus*.

a *With a significant postconceptional age X diagnosis interaction, estimated slopes are provided because the difference in means between SIDS cases and controls varies by age*.

b *Slope differs from zero, p < 0.05*.

### Hippocampal Features

We focused on three hippocampal features that were significantly more common in SIDS cases compared with controls (8); focal granule cell bilamination, clusters of immature cells in the subgranular layer, and single ectopic granule cells in the molecular layer of the DG. Figure 1 shows an example of these features while Table 3 shows the prevalence of these abnormalities in the original report (8) and in the SIDS analytic cohort from this report that have medullary 5-HT_1A_ data. Of note, the prevalence of clusters of immature cells in the subgranular layer was higher (73.2% vs. 53.5%, *p* = 0.04) in the current SIDS cases compared to the prevalence in the published cases (Table 3). This may be due to sampling variation. The prevalences of the other hippocampal features were similar for the Kinney et al. (8) and present study.

**Table 3:** Prevalence of hippocampal features of interest in Kinney et al. (8) and their prevalence in the analytic cohort of SIDS with available medullary 5-HT_1A_ binding data.

**Hippocampal feature**	**Published prevalence in control (8)**	Published prevalence in SIDS (8)^*^	**Prevalence in the analytic cohort of SIDS cases with medullary 5-HT_**1A**_ data**
Focal granule cell bilamination	7.7%; 95% CI 1.6–20.9% (3/39)	41.2%; 95% CI 32.1–50.8% (47/114)	56.1%; 95% CI 39.8–71.5% (23/41)
Clusters of immature cells in subgranular layer	10.3%; 95% CI 2.9–24.2% (4/39)	53.5%; 95% CI 43.9–62.9% (61/114)	73.2%; 95% CI 57.1–85.8% (30/41)
Single ectopic granule cells in molecular layer of dentate gyrus	33.3%; 95% CI 19.1–50.2% (13/39)	57.9%; 95% CI 48.3–67.1% (66/114)	63.4%; 95% CI 46.9–77.9% (26/41)

* *The prevalence of each hippocampal feature is significantly greater in SIDS than controls (p ≤ 0.01) (8). SIDS, sudden infant death syndrome; 5-HT, serotonin; CI, confidence interval*.

### Demographic Data of SIDS Analytic Cohort With Both Hippocampal Analysis and Medullary 5-HT_1A_ Binding

Demographic data for all SIDS cases in the analytic cohort are shown in Table 4. There were no significant differences in the demographics of the cases from the three datasets (Datasets 3, 4, and 5) (data not shown).

**Table 4:** Demographics of the SIDS analytic cohort with hippocampal assessment and 5-HT_1A_ binding.

	**Analytic cohort of SIDS cases with medullary 5-HT_1A_ analysis and hippocampal assessment Mean ± SD or *n* (%)**
*N*	41
Gestational age (wk)	38.7 ± 2.9
Postnatal age (wk)	16.3 ± 8.5
Postconceptional age (wk)	54.9 ± 8.1
Median (IQR) postconceptional age (wk)	53.0 (49.0, 59.0)
Prematurity (GA <37 weeks)	6 (15%)
Postmortem interval (hr)	19.7 ± 6.2
Male sex	25 (61%)
**Race/ethnicity**	
White	18 (46%)
Black	2 (5%)
Hispanic	12 (31%)
Other	7 (18%)
Unknown	2

*SIDS, sudden infant death syndrome; 5-HT, serotonin; wk, week; GA, gestational age; hr, hour; SD, Standard Deviation; %, percent; IQR, interquartile range*.

### Comparison of Mean 5-HT_1A_ Binding With the Presence or Absence of Specific Hippocampal Abnormalities

In the SIDS analytic cohort with both hippocampal analysis and 5-HT_1A_ binding data (*n* = 41), we addressed the hypothesis that the SIDS cases with a hippocampal abnormality will have lower medullary 5-HT_1A_ binding compared to SIDS cases without a hippocampal feature (Table 5). We found no difference in medullary 5-HT_1A_ binding levels in SIDS cases with or without granule cell bilamination (Table 5). There was no difference in medullary 5-HT_1A_ binding in 7 of 8 nuclei with or without clusters of immature cells in the subgranular layer. One exception, the raphe obscurus (RO), showed higher mean binding in SIDS cases with the hippocampal abnormality (*p* = 0.04) (Table 5). There was no difference in medullary 5-HT_1A_ binding in 6 of 8 nuclei with or without single ectopic granule cells in the molecular layer of the DG. In the HG and DMX, mean medullary 5-HT_1A_ binding was lower (*p* = 0.033 and 0.01, respectively) in SIDS cases with the hippocampal feature compared to SIDS cases without the hippocampal feature (Table 5).

**Table 5:** Medullary 5-HT_1A_ binding in SIDS cases with and without specific hippocampal abnormalities.

**Medullary Nucleus**		**Adjusted Mean 5-HT_1A_** **binding in fmol/mg** **±** **SE**		**Age- and dataset- adjusted *p*-value**
	**N Absent/Present**	**Hippocampal abnormality-ABSENT**	**Hippocampal abnormality-PRESENT**	
**FOCAL GRANULE CELL BILAMINATION**				
RO	16/23	38.67 ± 6.36	33.57 ± 5.06	0.49
GC	16/23	15.73 ± 2.27	14.58 ± 1.80	0.66
PGCL	16/23	12.80 ± 1.76	11.79 ± 1.40	0.62
IRZ	16/22	10.03 ± 1.44	8.75 ± 1.16	0.45
ARC	10/15	3.88 ± 1.27	4.80 ± 0.95	0.51
HG	15/16	7.47 ± 1.17	7.21 ± 1.05	0.86
DMX	14/13	8.08 ± 1.05	7.64 ± 1.13	0.78
NTS	15/16	9.13 ± 1.09	9.05 ± 0.98	0.95
**CLUSTERS OF IMMATURE CELLS IN SUBGRANULAR LAYER**				
RO	9/30	21.82 ± 7.67	39.34 ± 4.49	**0.04**
GC	9/30	11.97 ± 2.83	15.87 ± 1.65	0.21
PGCL	9/30	9.70 ± 2.19	12.87 ± 1.29	0.20
IRZ	9/29	7.80 ± 1.82	9.65 ± 1.08	0.36
ARC	5/20	4.65 ± 1.62	4.49 ± 0.90	0.92
HG	8/23	6.52 ± 1.60	7.57 ± 0.92	0.57
DMX	7/20	7.65 ± 1.64	7.94 ± 0.89	0.88
NTS	8/23	7.76 ± 1.48	9.48 ± 0.85	0.30
**SINGLE ECTOPIC GRANULE CELLS IN MOLECULAR LAYER OF DG**				
RO	14/25	41.24 ± 6.32	31.80 ± 5.14	0.22
GC	14/25	15.13 ± 2.29	14.92 ± 1.86	0.94
PGCL	14/25	12.56 ± 1.78	11.91 ± 1.45	0.76
IRZ	14/24	10.49 ± 1.43	8.41 ± 1.18	0.24
ARC	10/15	4.08 ± 1.13	4.86 ± 1.05	0.57
HG	13/18	9.08 ± 1.08	5.98 ± 0.96	**0.03**
DMX	5/11	10.17 ± 1.06	6.27 ± 0.89	**0.01**
NTS	13/18	10.55 ± 1.04	7.97 ± 0.92	0.06

*SIDS, sudden infant death syndrome; 5-HT, serotonin; RO, raphe obscurus; GC, gigantocellularis; PGCL, paragigantocellularis lateralis; IRZ, intermediate reticular zone; ARC, arcuate nucleus; HG, hypoglossal nucleus; DMX, dorsal motor nucleus of the vagus; NTS, nucleus of the solitary tract; SE, standard error. The numbers (N) of SIDS cases with the presence or absence of the hippocampal feature are given in the second column. The total N varies due to the fact that not every SIDS case had binding in every nucleus. Significant p-values (p < 0.05) are bolded*.

### Association Between the Presence of a Hippocampal Abnormality and Low 5-HT_1A_ Binding

We addressed the hypothesis that SIDS cases with the lowest medullary 5-HT_1A_ binding have a higher prevalence of hippocampal abnormalities compared to SIDS cases with higher binding. We rationalized that if there is an association between a hippocampal feature and low medullary 5-HT_1A_ binding, there would be a higher prevalence of the feature in the SIDS cases with the lowest binding. We saw no difference in the prevalence of hippocampal abnormalities in SIDS cases with the lowest binding (Q1) compared to SIDS cases defined as having higher binding (Q2-Q4). This was true for all hippocampal abnormalities and all medullary nuclei including a composite measure representing low binding in at least two nuclei (Table 6).

**Table 6:** Estimated age-adjusted prevalence of hippocampal abnormality in SIDS cases with low (first quartile) vs. higher 5-HT_1A_ binding (quartiles 2–4).

**Medullary**	**N Q1/Q2–Q4**	**% with hippocampal**		**Age-adjusted *p*-value**
**nucleus**		**feature** **±** **SE**		
		**Q1**	**Q2–Q4**	
**FOCAL GRANULE CELL BILAMINATION**				
Composite^*^	16/25	66 ± 12	50 ± 10	0.35
RO	10/29	61 ± 16	59 ± 9	0.90
GC	10/29	43 ± 16	65 ± 9	0.25
PGCL	11/28	58 ± 16	60 ± 9	094
IRZ	10/28	50 ± 16	61 ± 9	0.54
ARC	7/18	44 ± 19	66 ± 11	0.31
HG	9/22	55 ± 17	50 ± 11	0.81
DMX	8/19	67 ± 17	40 ± 12	0.25
NTS	9/22	45 ± 17	54 ± 11	0.62
**CLUSTERS OF IMMATURE CELLS IN SUBGRANULAR LAYER**				
Composite^*^	16/25	74 ± 12	76 ± 9	0.90
RO	10/29	73 ± 15	81 ± 8	0.59
GC	10/29	68 ± 16	83 ± 7	0.34
PGCL	11/28	71 ± 15	82 ± 7	0.45
IRZ	10/28	60 ± 17	85 ± 7	0.14
ARC	7/18	89 ± 11	79 ± 10	0.56
HG	9/22	67 ± 17	81 ± 9	0.42
DMX	8/19	84 ± 13	72 ± 11	0.52
NTS	9/22	69 ± 16	80 ± 9	0.53
**SINGLE ECTOPIC GRANULE CELLS IN MOLECULAR LAYER OF THE**				
**DENTATE GYRUS**				
Composite^*^	16/25	68 ± 13	62 ± 10	0.71
RO	10/29	73 ± 15	62 ± 10	0.57
GC	10/29	57 ± 17	68 ± 10	0.56
PGCL	11/28	72 ± 15	63 ± 10	0.62
IRZ	10/28	60 ± 16	65 ± 9	0.78
ARC	7/18	46 ± 19	66 ± 12	0.36
HG	9/22	79 ± 14	50 ± 11	0.17
DMX	8/19	89 ± 11	43 ± 14	0.08
NTS	9/22	81 ± 14	49 ± 12	0.14

*Estimates are adjusted for post-conceptional age and classification of low binding is performed within dataset. SIDS, sudden infant death syndrome; 5-HT, serotonin; RO, raphe obscurus; GC, gigantocellularis; PGCL, paragigantocellularis lateralis; IRZ, intermediate reticular zone; ARC, arcuate nucleus; HG, hypoglossal nucleus; DMX, dorsal motor nucleus of the vagus; NTS, nucleus of the solitary tract; SE, standard error. The numbers (N) of SIDS cases in Q1 and Q2–Q4 are given in the second column. The total N varies due to the fact that not every SIDS case had binding in every nucleus*.

* *Composite measure is an indicator for a case having low 5-HT_1A_ binding (Q1) in at least 2 nuclei*.

### Analyses of SIDS Subsets as Defined by the Presence or Absence of Low Medullary 5-HT_1A_ Binding and Hippocampal Features

**Figure 4:**
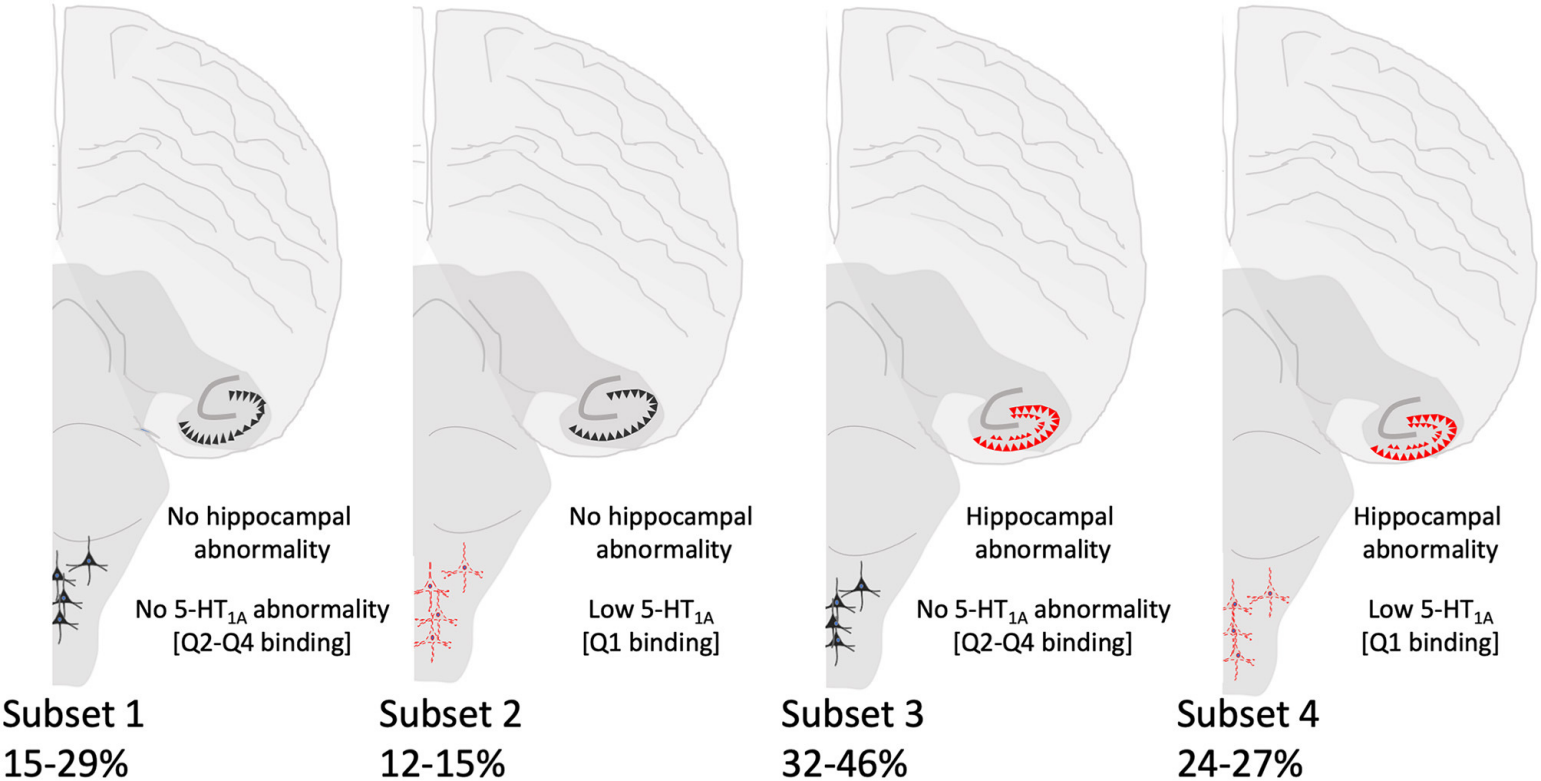
Summary of SIDS subsets based on hippocampal and brainstem (5-HT_1A_) abnormalities. Hippocampal morphological and brainstem 5-HT_1A_ abnormalities are depicted in red. The percentages of SIDS cases falling within each subset are shown and vary depending on the hippocampal feature.

Although we did not find significant associations between low medullary 5-HT_1A_ binding (defined as binding in the lowest quartile) and hippocampal abnormalities (Table 6), there are cases within the cohort that exhibit both lesions [Subset 4] (Figure 4). Table 7 shows clinical and risk factor data associated with SIDS subsets based on the presence or absence of medullary 5-HT_1A_ binding in the lowest quartile of binding (Q1) with and without the presence of focal granule cell bilamination. There were 10 SIDS cases (10/41, 24%) that showed both low medullary 5-HT_1A_ binding and DG bilamination [Subset 4]. There was no significant difference in PCA, gestational age (GA), male sex, illness 24–48 h prior to death, body position (prone), face position (face down or face covered), prevalence of bedsharing, or sleep site across the different groups. There was a significant difference (*p* = 0.007) in the prevalence of premature birth (birth <37 gestational weeks), with the highest prevalence of premature birth in the SIDS subset with low 5-HT_1A_ binding only (50%) (Table 7) [Subset 2]. There were no premature infants in the SIDS subset with focal granule cell bilamination, with or without low medullary 5-HT_1A_ binding [Subset 3 and 4, respectively]. There was no difference in reported prenatal alcohol exposure. There was a higher prevalence of prenatal smoking (60%) in the subset with medullary 5-HT_1A_ abnormalities only [Subset 2] compared with the other three subsets (0–33% prenatal smoking), but this comparison was not statistically significant (*p* = 0.10). In addition to the clinical and risk factors listed, we also examined the prevalence of the following factors: history of illness 1 week prior to death, position to sleep, position found, prenatal exposure to selective serotonin reuptake inhibitors (SSRIs), complications of pregnancy, complications of labor, complications of delivery, complications of the postnatal period, and minor congenital abnormalities. There were no statistical differences found with these clinical features among the SIDS subsets (data not shown).

**Table 7:** Clinical and risk factor profile by medullary 5-HT_1A_ binding and hippocampal feature (focal granule cell bilamination) status.

**Clinical feature/risk factor**	**5-HT_1A_ binding within Q2–Q4 without hippocampal feature**	**Low (Q1) 5-HT_1A_ binding only**	**Hippocampal feature Only**	**Low (Q1) 5-HT_1A_ binding with hippocampal feature**	***p*-value**
	**[Subset 1]**	**[Subset 2]**	**[Subset 3]**	**[Subset 4]**	
*N*	12 (29%)	6 (15%)	13 (32%)	10 (24%)	
Postconceptional age (wk)	54.7 ± 6.8	61.0 ± 11.2	53.0 ± 8.3	54.2 ± 6.4	0.24
Gestational age (wk)	37.8 ± 4.4	37.5 ± 3.6	39.3 ± 1.1	39.7 ± 0.9	0.10
Prematurity	3 (25%)	3 (50%)	0 (0%)	0 (0%)	**0.007**
Male sex	7 (58%)	4 (67%)	9 (69%)	5 (50%)	0.82
Illness 24–48 h prior to death (*N* = 12; 6; 13; 9)	2 (17%)	2 (33%)	4 (31%)	4 (44%)	0.59
Found prone (*N* = 12; 4; 12; 8)	5 (42%)	2 (50%)	6 (50%)	7 (88%)	0.19
Face down or Face covered (*N* = 9; 3; 9; 7)	4 (36%)	2 (18%)	1 (9%)	4 (36%)	0.18
Bed Sharing	3 (25%)	1 (17%)	4 (31%)	2 (20%)	0.96
Smoking (*N* = 6; 5; 12; 6)	0	3 (60%)	2 (17%)	2 (33%)	0.10
Alcohol (*N* = 6; 4; 9; 7)	0	0	0	1 (14%)	0.65
Sleep site (*N* = 12; 6; 11; 10)					0.18
Crib	7 (58%)	4 (67%)	2 (18%)	5 (50%)	
Adult bed	4 (33%)	1 (17%)	8 (73%)	4 (40%)	
Sofa	0	0	0	0	
Car seat	0	0	1 (9%)	0	
Other	1 (8%)	1 (17%)	0 (0%)	1 (10%)	

*5-HT, serotonin; Q, quartile; N, number; wk, week. Information on some risk factors was not available for every case. The N's shown under the risk factor in the first column indicate the number of cases in each subset with information available when missing data were present. Significant p-values (p < 0.05) are bolded*.

We performed similar analyses on SIDS subsets with and without hippocampal clusters of immature cells in the subgranular layer of the hippocampus (Table 8). Mean PCA was higher in the SIDS subset without either lesion (Subset 1) (*p* = 0.04) (Table 8). This reflects a higher postnatal age in this subset, given that gestational age is not different. Cases from this same group were also more likely to have been sleeping somewhere besides the crib (*p* = 0.03). There were no significant differences among the subsets in prenatal exposures to SSRIs, alcohol, and smoking.

**Table 8:** Clinical and risk factor profile by medullary 5-HT_1A_ binding and hippocampal feature (clusters of immature cells in the subgranular layer).

**Clinical feature/risk Factor**	**5-HT_1A_ binding within Q2–Q4 without hippocampal feature**	**Low (Q1) 5-HT_1A_ binding only**	**Hippocampal feature only**	**Low (Q1) 5-HT_1A_ binding with hippocampal feature**	***p*-value**
	**[Subset 1]**	**[Subset 2]**	**[Subset 3]**	**[Subset 4]**	
*N*	6 (15%)	5 (12%)	19 (46%)	11 (27%)	
Postconceptional age (wk)	61.3 ± 9.3	57.1 ± 13.6	51.4 ± 5.1	56.5 ± 6.6	**0.04**
Gestational age (wk)	38.8 ± 2.4	37.7 ± 3.5	38.5 ± 3.4	39.4 ± 1.9	0.77
Prematurity	1 (17%)	2 (40%)	2 (11%)	1 (10%)	0.33
Male sex	4 (67%)	4 (80%)	12 (63%)	5 (45%)	0.60
Found prone (*N* = 6; 3; 18;9)	2 (33%)	2 (67%)	9 (50%)	7 (78%)	0.35
Face down or Face covered (*N* = 5; 3; 13;7)	0 (0%)	2 (18%)	5 (45%)	4 (36%)	0.18
Bed Sharing	1 (17%)	0 (0%)	6 (32%)	3 (27%)	0.67
Illness 24–48 h prior to death (*N* = 6; 4; 19; 11)	1 (17%)	1 (25%)	5 (26%)	5 (45%)	0.65
Smoking (*N* = 4; 3; 14; 8)	0	2 (67%)	2 (14%)	3 (38%)	0.14
Alcohol (*N* = 4; 2; 11; 9)	0	0	0	1 (11%)	0.58
Sleep site (*N* = 6; 5; 17; 11)					**0.03**
Crib	1 (17%)	3 (60%)	8 (47%)	6 (55%)	
Adult bed	3 (50%)	0 (0%)	9 (53%)	5 (45%)	
Sofa	0	0	0	0	
Car seat	1 (17%)	0	0	0	
Other	1 (17%)	2 (40%)	0	0	

*5-HT, serotonin; Q, quartile; N, number; wk, week. Information on some risk factors was not available for every case. The N's given under the risk factor in the first column indicate the number of cases in each subset with information available when missing data were present. Significant p-values (p < 0.05) are bolded*.

In the analysis of SIDS subsets with and without single ectopic granule cells in the molecular layer of the DG (Table 9), there was a borderline significant difference in PCA (*p* = 0.05) and the face position (face down or face covered) (*p* = 0.09). There was a difference in prenatal smoking (*p* = 0.02) with the highest prevalence of smoking (71%) in the cases with both hippocampal and brainstem 5-HT_1A_ abnormalities [Subset 4]. There were no significant differences amongst the groups in any other clinical or risk factors analyzed (data not shown).

**Table 9:** Clinical and risk factor profile by medullary 5-HT_1A_ binding and hippocampal feature (single ectopic granule cells in the molecular layer of the dentate gyrus) status.

**Clinical feature/risk factor**	**5-HT_1A_ binding within Q2-Q4 without hippocampal feature**	**Low (Q1) 5-HT_1A_ binding only**	**Hippocampal feature only**	**Low (Q1) 5-HT_1A_ binding with hippocampal feature**	***p*-value**
	**[Subset 1]**	**[Subset 2]**	**[Subset 3]**	**[Subset 4]**	
*N*	9 (22%)	6 (15%)	16 (39%)	10 (24%)	
Postconceptional age (wk)	56.5 ± 9.4	62.3 ± 8.9	52.3 ± 5.9	53.4 ± 7.3	0.05
Gestational age (wk)	39.5 ± 2.1	39.0 ± 2.5	38.1 ± 3.6	38.8 ± 2.6	0.67
Prematurity	1 (11%)	1 (17%)	2 (13%)	2 (20%)	0.93
Male sex	3 (33%)	2 (33%)	6 (38%)	5 (50%)	0.89
Illness 24–48 h prior to death (*N* = 9; 6; 16; 9)	3 (33%)	1 (17%)	3 (19%)	5 (56%)	0.26
Found prone (*N* = 8; 4; 16; 8)	2 (25%)	4 (100%)	9 (57%)	5 (63%)	0.12
Face down or Face covered (*N* = 5; 3; 13; 7)	0	1 (9%)	5 (45%)	5 (45%)	0.09
Bed Sharing	2 (22%)	1 (17%)	5 (31%)	2 (20%)	0.92
Smoking (*N* = 7; 4; 11; 7)	1 (14%)	0	1 (9%)	5 (71%)	**0.02**
Alcohol (*N* = 5; 4; 10; 7)	0	0	0	1 (14%)	0.62
Sleep site (*N* = 7;6;16;10)					0.32
Crib	2 (29%)	4 (67%)	7 (44%)	5 (50%)	
Adult bed	3 (43%)	2 (33%)	9 (56%)	3 (30%)	
Sofa	0	0	0	0	
Car seat	1 (14%)	0	0	0	
Other	1 (14%)	0	0	2 (20%)	

*5-HT, serotonin; Q, quartile; N, number; wk, week. Information on some risk factors was not available for every case. The N's given under the risk factor in the first column indicate the number of cases in each subset with information available when missing data were present. Significant p-values (p < 0.05) are bolded*.

## Discussion

We used a combined analytic cohort of SIDS cases from our laboratory to statistically address the hypothesis that hippocampal abnormalities and medullary 5-HT_1A_ abnormalities are associated, with one dependent on the presence of the other. We hypothesized that evidence supporting a dependent relationship between the two lesions would be demonstrated in either (1) decreased medullary binding in the presence of one or more hippocampal lesions, (2) an increased prevalence of hippocampal abnormalities in the cases with the lowest medullary binding, and/or (3) a uneven distribution of cases across the 4 designated subsets with an increased number of SIDS cases with both abnormalities present compared to SIDS cases with only one abnormality. While our resulting data largely support the important observation that our overarching hypothesis is not true, they also highlight the complexity of SIDS etiology. Below we discuss our findings, the limitations of the methods, and contribution of the data to our understanding of SIDS pathology.

### Hippocampal-Brainstem Relationship

Our analyses focused on three hippocampal features reported by Kinney et al., to be significantly present in SIDS infants compared to controls (8). The prevalence of these features, particularly granule cell bilamination, among different SIDS cohorts has varied (14, 15) as has the reported specificity of the finding to pathology in pediatric cohorts including SIDS and sudden unexplained death in childhood (9, 10, 12, 14, 29–32). Differences in the statistical significance of these hippocampal features likely reflect cohort size, availability and definitions of controls, differences in and availability of consistent hippocampal levels, and differences among neuropathological assessments. Despite differences among studies, our combined analytic cohort presented a unique opportunity to examine potential relationships between the observations in our laboratory of hippocampal abnormalities and medullary 5-HT_1A_ deficiencies. Using statistical methods, we were largely unable to detect the hypothesized relationships. Nonetheless, we did see significant differences in medullary 5-HT_1A_ values when comparing cases with and without clusters of immature cells in the subgranular layer (increased binding in the RO of cases with the hippocampal abnormality) and when comparing cases with and without single ectopic granular cells in the molecular layer of the DG (decreased binding in the HG and DMX of cases with the hippocampal abnormality). The biological significance of these findings is unknown however, particularly given the lack of statistical significance when we examined the prevalence of the hippocampal lesions in cases with low 5-HT_1A_ binding at these medullary sites.

Our original hypothesis that medullary and hippocampal findings in SIDS infants coexist was based partially on the known trophic role of 5-HT during development in neuronal migration and neurogenesis, including in the dentate gyrus of the hippocampus (22–25). While 5-HT present in the hippocampus during hippocampal development is thought to mainly derive from rostral 5-HT groups in the midbrain and pons (33, 34), connectivity between medullary 5-HT nuclei and limbic structures including hippocampus have been shown in the human (27), suggesting a potential additional role for caudal 5-HT groups in hippocampal development. We speculated that abnormalities in the hippocampus in SIDS reflect proliferation and/or migration defects and are due to defective or deficient brainstem 5-HT innervation of the hippocampus, including innervation of the hippocampal Cajal Retzius cells that produce the reelin during development and regulate neuronal migration (24). In addition to potential trophic implications of an abnormal serotonergic system on hippocampal formation, we also considered a potential implication of an abnormal hippocampus on the medullary 5-HT system. Our findings in the hippocampus represent a putative morphological marker of an impaired central homeostatic network involving the limbic system (including hippocampus), brainstem and forebrain (35). An instability in limbic regions, potentially resulting in abnormal seizure-like electrical discharges, could propagate to the medullary regions involved in breathing and/or autonomic function decreasing activity of the 5-HT neurons. The effect of seizure on 5-HT neuronal activity in the medulla during and after seizure activity has been shown in rat models (26) and patients with temporal lobe epilepsy exhibit decreased binding to 5-HT_1A_ receptors within the midbrain raphe (36, 37). Given this, we also postulated a scenario where an abnormal hippocampus and hippocampal electrical discharge, either acutely at the time of death or intermittently during the postnatal period, could lead to abnormal medullary 5-HT neuronal activity in SIDS infants.

In our analysis of SIDS subsets (summarized in Figure 4), our assessment of available clinical and risk factor data shows no distinct profile associated with SIDS cases presenting with both hippocampal and medullary abnormalities, SIDS cases with neither hippocampal nor medullary abnormalities, nor SIDS cases presenting at autopsy with abnormalities at only one site (hippocampus or medulla). Interestingly, in our analysis of SIDS subsets with and without low medullary 5-HT_1A_ binding and single ectopic granule cells in the molecular layer of the DG (Table 9), we showed a significantly higher rate of prenatal exposure to smoking in the subset of cases with both the brainstem and the hippocampal abnormality. Prenatal smoking is a known risk factor for SIDS and has been related to lower 5-HT_1A_ receptor expression in medullary nuclei of postmortem infants (SIDS and controls) compared with cases with no prenatal smoking history (7). Rodent and primate models of prenatal nicotine exposure also show an effect of prenatal nicotine exposure on the 5-HT_1A_ receptor, albeit with increased 5-HT_1A_ receptor expression (38) and binding (39), respectively. Relative to hippocampal development, prenatal nicotine exposure has effects on hippocampal neuronal signaling and function (40) as well as morphological indices [reviewed in (41)]. Our findings related to this subset of SIDS cases suggest a developmental relationship or connectivity between the medullary and hippocampal entities that is affected more so by prenatal exposure than is either entity alone. The number of cases with exposure information is relatively small and therefore this result should be considered as hypothesis-generating (see Limitations below). In our analysis of SIDS subsets with and without low medullary 5-HT_1A_ binding and focal granule cell bilamination, there was a higher rate of prematurity in the SIDS subset with low medullary 5-HT_1A_ binding only (Table 7). Prematurity is a known risk factor for SIDS (42) and vulnerability within this group related to deficits in the medullary 5-HT system is of interest and warrants further study. In this same analysis of SIDS subsets with and without low medullary 5-HT_1A_ and clusters of immature cells in the subgranular layer (Table 8), we showed a significant difference in sleep site with a higher proportion of cases with neither hippocampal or medullary abnormalities sleeping in sites other than the crib (e.g., adult bed). Given the low numbers included in this analysis, the significance is unknown. It may, however, reflect a need for an increased burden of SIDS risks factors (sleeping in an adult bed) to precipitate death in cases without these abnormalities.

In our analysis of SIDS subsets, the number of cases (~25%) with both hippocampal and 5-HT_1A_ abnormalities is of interest. We postulate that in these cases, the hippocampal and brainstem abnormalities may be related, either via mechanisms suggested above or in ways related to an unknown common cause lying upstream of both. In cases displaying only one abnormality, we cannot rule out the possibility that the other abnormality would present itself had the infant lived long enough. Finally, in cases with neither abnormality, the question remains as to the underlying pathogenesis. Death in these cases may be related to other intrinsic (e.g., genetic) or extrinsic (e.g. environmental) risk factors alone or in combination postulated to play a role in SIDS [reviewed in (43)].

In our analyses, we focus on brainstem abnormalities as determined by binding deficiencies in the 5-HT_1A_ receptor in the medulla only. We cannot rule out the possibility that hippocampal abnormalities co-exist with potential 5-HT abnormalities in rostral brainstem structures (pontine or midbrain). Of note, in addition to medullary 5-HT_1A_ abnormalities, we have also observed abnormalities in other 5-HT indices in the medulla including a defect in binding to ^3^H- lysergic acid diethylamide (LSD), a much broader 5-HT receptor ligand (1, 3), a deficiency in 5-HT levels as determined by high performance liquid chromatography (HPLC) (5), and an increased number of neurons expressing tryptophan hydroxylase 2 (TPH2) [rate determining enzyme in 5-HT production] as determined by immunocytochemistry (4). Thus, we cannot rule out the possibility that hippocampal abnormalities co-exist with other 5-HT abnormalities in rostral or caudal brainstem structures. Overlap between cases to date with hippocampal data and medullary measures of 5-HT level and TPH2 cell number is insufficient to look for associations as we have done here.

### Medullary 5-HT_1A_ Abnormalities in the Combined Cohort

In addition to new data discussed above on the relationship between hippocampus and medullary abnormalities, it is important also to emphasize the medullary 5-HT_1A_ data in the full combined cohort [published and unpublished] of SIDS and controls (Table 2, Figure 3). In this combined cohort, we have confirmed published deficiencies in 5-HT_1A_ binding in multiple nuclei of the rostral and caudal medulla, including nuclei containing 5-HT neurons (RO, GC, PGCL, and IRZ) and nuclei containing 5-HT projections (HG and NTS). These data support the robustness of the published 5-HT_1A_ binding abnormalities in SIDS. Abnormalities in binding include overall decreased binding in SIDS compared to controls (GC and NTS) and significant age vs. diagnosis interactions (RO, PGCL, IRZ, and HG). The latter finding, first reported in Duncan et. al. (5), shows a decreased binding with age in the SIDS cases only. This decrease in binding potentially reflects a dynamic change with age due to instability in binding or compensation over time in response to some other factor or developmental abnormality. Alternatively, it may reflect the possibility that the most vulnerable infants (lowest 5-HT_1A_ binding) are still susceptible at an older age or that infants with a greater deficiency live longer, potentially avoiding for a longer period of time the external stressors that we hypothesize trigger sudden death.

### Limitations of Methods

To address the relationship between hippocampal and brainstem 5-HT_1A_ abnormalities, we have utilized a analytic cohort of SIDS cases only. Overlapping hippocampal and brainstem data exist for only 5–9 control cases thus limiting our ability to utilize controls for comparison. Within the SIDS analytic cohort, we have defined low 5-HT_1A_ binding as cases in the lowest quartile (lowest 25%) of binding compared to SIDS cases with binding in all other quartiles (26–100%). We cannot exclude the possibility that hippocampal abnormalities statistically associate with more subtle 5-HT_1A_ deficiencies—that is, SIDS cases falling within the second quartile of binding (25–50%). Mostly non-significant differences in medullary 5-HT_1A_ binding with or without hippocampal dysmorphology suggests however, that this is not the case (Table 5). We also cannot rule out that hippocampal abnormalities associate with medullary 5-HT_1A_ abnormalities defined as low based on controls, an analysis that we could not do due to reasons discussed. Within our SIDS analytic cohort, medullary 5-HT_1A_ data were not available for all medullary nuclei. Thus, in our analysis of SIDS subsets (Tables 7–9), our designations of subsets based on low 5-HT_1A_ binding in two or more medullary nuclei have been given without knowledge in some cases of binding in all nuclei. Our receptor binding analyses over 14 years covered three independent datasets. In the binding experiments, we utilize radioactive standards, which normalize the data across experiments. However, over the three different datasets, there were small but significant differences, specifically with Dataset 5 compared with Datasets 3 and 4. This difference was statistically adjusted for in our final analysis but is included in the limitations given the unavoidable nature of experimental variation over such a long period of time. Finally in our analysis of clinical and risk factor data, we report only on what is available in the autopsy and investigative reports. While we analyzed the data for differences in prenatal exposures, we consider these data with caution. The information that is not available is likely missing at random and thus not incurring bias into the comparisons performed. However, the number of cases where exposure information is provided is relatively low and the exposure data that we do have on the cases is general (yes/no) with little information about quantity of exposure or when the exposure occurred (e.g, first, second, or third trimester).

### Implications of Independent Hippocampal and Medullary 5-HT_1A_ Brainstem Lesions

Our data support that the presence of the three hippocampal features identified previously as increased in SIDS infants (8) is not strictly dependent on the presence of abnormalities in medullary 5-HT_1A_ binding. Whether these lesions reflect two independent diseases or one disease with minor differences in pathological phenotypes remains unknown. The former supports a heterogenous etiology of SIDS while the latter suggests a common disease process with mechanisms affecting different nodes within the integrated central homeostatic network. Given the number of SIDS cases with and without one or both of the lesions, our data support the heterogenous nature of SIDS with different vulnerabilities in different infants. Hypotheses regarding biological or mechanistic relationship(s) between different vulnerabilities in SIDS and the means by which risk factors intersect these vulnerabilities to increase susceptibility to sudden death remain critical.

## Data Availability Statement

Requests to access these datasets should be directed to robin.haynes@childrens.harvard.edu.

## Author Contributions

RH had full access to all the data in the study and takes responsibility for the integrity of the data and the accuracy of the data analysis. RH, HK, and LS: concept and design and drafting of the manuscript. RH, HK, EH, JD, MR, FT, DA, SA, JC, HK, MH, RG, and LS: critical revision of the manuscript for important intellectual content. LS: statistical analysis. RH, HK, and RG: obtained funding. EH, JD, MR, FT, RG, SA, DA, JC, HK, and MH: administrative, technical, or material support. RH and HK: supervision. All authors contributed to the article and approved the submitted version.

## Funding

This work was funded by the National Institute of Child Health and Development (R01-HD090064, PO1-HD036379), CJ Foundation for SIDS, Cooper Trewin Brighter Days Fund, River's Gift, Evelyn Deborah Barrett Fellowship for SIDS Research, Marley J. Cherella Fellowship for SIDS Research, First Candle/SIDS Alliance, CJ Murphy Foundation for Solving the Puzzle of SIDS, Barrett Tallman Memorial Fund, Florida SIDS Alliance, Jacob Neil Boger Foundation for SIDS, Jason Lutz SIDS Foundation, Three Butterflies Foundation, Bennett C. Endres Fellowship, The Family of Lyla Heffernan, and Robert's Program on Sudden Unexpected Death in Pediatrics. We thank the IDDRC Core, funded by NIH U54 HD090255.

## Conflict of Interest

FT is employed by HealthCore, Inc. The remaining authors declare that the research was conducted in the absence of any commercial or financial relationships that could be construed as a potential conflict of interest.

## Publisher's Note

All claims expressed in this article are solely those of the authors and do not necessarily represent those of their affiliated organizations, or those of the publisher, the editors and the reviewers. Any product that may be evaluated in this article, or claim that may be made by its manufacturer, is not guaranteed or endorsed by the publisher.

## References

[1] Panigrahy A Filiano J Sleeper LA Mandell F Valdes-Dapena M Krous HF. Decreased serotonergic receptor binding in rhombic lip-derived regions of the medulla oblongata in the sudden infant death syndrome. J Neuropathol Exp Neurol. (2000) 59:377–84. 10.1093/jnen/59.5.377 10888367

[2] Kinney HC Filiano JJ White WF. Medullary serotonergic network deficiency in the sudden infant death syndrome: review of a 15-year study of a single dataset. J Neuropathol Exp Neurol. (2001) 60:228–47. 10.1093/jnen/60.3.228 11245208

[3] Kinney HC Randall LL Sleeper LA Willinger M Belliveau RA Zec N. Serotonergic brainstem abnormalities in Northern Plains Indians with the sudden infant death syndrome. J Neuropathol Exp Neurol. (2003) 62:1178–91. 10.1093/jnen/62.11.1178 14656075

[4] Paterson DS Trachtenberg FL Thompson EG Belliveau RA Beggs AH Darnall R. Multiple serotonergic brainstem abnormalities in sudden infant death syndrome. JAMA. (2006) 296:2124–32. 10.1001/jama.296.17.2124 17077377

[5] Duncan JR Paterson DS Hoffman JM Mokler DJ Borenstein NS Belliveau RA. Brainstem serotonergic deficiency in sudden infant death syndrome. JAMA. (2010) 303:430–7. 10.1001/jama.2010.45 20124538 PMC3242415

[6] Barnes NM Sharp T. A review of central 5-HT receptors and their function. Neuropharmacology. (1999) 38:1083–152. 10.1016/S0028-3908(99)00010-6 10462127

[7] Machaalani R Say M Waters KA. Serotoninergic receptor 1A in the sudden infant death syndrome brainstem medulla and associations with clinical risk factors. Acta Neuropathol. (2009) 117:257–65. 10.1007/s00401-008-0468-x 19052756

[8] Kinney HC Cryan JB Haynes RL Paterson DS Haas EA Mena OJ. Dentate gyrus abnormalities in sudden unexplained death in infants: morphological marker of underlying brain vulnerability. Acta Neuropathol. (2015) 129:65–80. 10.1007/s00401-014-1357-0 25421424 PMC4282685

[9] Kinney HC Armstrong DL Chadwick AE Crandall LA Hilbert C Belliveau RA. Sudden death in toddlers associated with developmental abnormalities of the hippocampus: a report of five cases. Pediatr Dev Pathol. (2007) 10:208–23. 10.2350/06-08-0144.1 17535090

[10] Kinney HC Chadwick AE Crandall LA Grafe M Armstrong DL Kupsky WJ. Sudden death, febrile seizures, and hippocampal and temporal lobe maldevelopment in toddlers: a new entity. Pediatr Dev Pathol. (2009) 12:455–63. 10.2350/08-09-0542.1 19606910 PMC3286023

[11] Rodriguez ML McMillan K Crandall LA Minter ME Grafe MR Poduri A. Hippocampal asymmetry and sudden unexpected death in infancy: a case report. Forensic Sci Med Pathol. (2012) 8:441–6. 10.1007/s12024-012-9367-5 22864821 PMC3897269

[12] Hefti MM Cryan JB Haas EA Chadwick AE Crandall LA Trachtenberg FL. Hippocampal malformation associated with sudden death in early childhood: a neuropathologic study: part 2 of the investigations of The San Diego SUDC Research Project. Forensic Sci Med Pathol. (2016) 12:14–25. 10.1007/s12024-015-9731-3 26782962

[13] Hefti MM Kinney HC Cryan JB Haas EA Chadwick AE Crandall LA. Sudden unexpected death in early childhood: general observations in a series of 151 cases: part 1 of the investigations of the San Diego SUDC Research Project. Forensic Sci Med Pathol. (2016) 12:4–13. 10.1007/s12024-015-9724-2 26782961 PMC4752958

[14] Kinney HC Poduri AH Cryan JB Haynes RL Teot L Sleeper LA. Hippocampal formation maldevelopment and sudden unexpected death across the pediatric age spectrum. J Neuropathol Exp Neurol. (2016) 75:981–97. 10.1093/jnen/nlw075 27612489 PMC6281079

[15] Kon FC Vazquez RZ Lang A Cohen MC. Hippocampal abnormalities and seizures: a 16-year single center review of sudden unexpected death in childhood, sudden unexpected death in epilepsy and SIDS. Forensic Sci Med Pathol. (2020) 16:423–34. 10.1007/s12024-020-00268-7 32712908

[16] Houser CR. Granule cell dispersion in the dentate gyrus of humans with temporal lobe epilepsy. Brain Res. (1990) 535:195–204. 10.1016/0006-8993(90)91601-C 1705855

[17] Armstrong DD. The neuropathology of temporal lobe epilepsy. J Neuropathol Exp Neurol. (1993) 52:433–43. 10.1097/00005072-199309000-00001 8360697

[18] Armstrong DD. Epilepsy-induced microarchitectural changes in the brain. Pediatr Dev Pathol. (2005) 8:607–14. 10.1007/s10024-005-0054-3 16333693

[19] Blumcke I Kistner I Clusmann H Schramm J Becker AJ Elger CE. Towards a clinico-pathological classification of granule cell dispersion in human mesial temporal lobe epilepsies. Acta Neuropathol. (2009) 117:535–44. 10.1007/s00401-009-0512-5 19277686

[20] Harper RM. State-related physiological changes and risk for the sudden infant death syndrome. Aust Paediatr J. (1986) 22(Suppl. 1):55–8. 3790006

[21] Richerson GB Buchanan GF. The serotonin axis: shared mechanisms in seizures, depression, and SUDEP. Epilepsia. (2011) 52(Suppl. 1):28–38. 10.1111/j.1528-1167.2010.02908.x 21214537 PMC3052632

[22] Whitaker-Azmitia PM. Role of serotonin and other neurotransmitter receptors in brain development: basis for developmental pharmacology. Pharmacol Rev. (1991) 43:553–61. 10.1007/978-3-0348-7259-1_5 1663620

[23] Gaspar P Cases O Maroteaux L. The developmental role of serotonin: news from mouse molecular genetics. Nat Rev Neurosci. (2003) 4:1002–12. 10.1038/nrn1256 14618156

[24] Janusonis S Gluncic V Rakic P. Early serotonergic projections to Cajal-Retzius cells: relevance for cortical development. J Neurosci. (2004) 24:1652–9. 10.1523/JNEUROSCI.4651-03.2004 14973240 PMC6730467

[25] Daubert EA Condron BG. Serotonin: a regulator of neuronal morphology and circuitry. Trends Neurosci. (2010) 33:424–34. 10.1016/j.tins.2010.05.005 20561690 PMC2929308

[26] Zhan Q Buchanan GF Motelow JE Andrews J Vitkovskiy P Chen WC. Impaired Serotonergic Brainstem Function during and after Seizures. J Neurosci. (2016) 36:2711–22. 10.1523/JNEUROSCI.4331-15.2016 26937010 PMC4879214

[27] Edlow BL McNab JA Witzel T Kinney HC. The Structural Connectome of the Human Central Homeostatic Network. Brain Connect. (2016) 6:187–200. 10.1089/brain.2015.0378 26530629 PMC4827322

[28] Kinney HC Haynes RL. The serotonin brainstem hypothesis for the sudden infant death syndrome. J Neuropathol Exp Neurol. (2019) 78:765–79. 10.1093/jnen/nlz062 31397480 PMC6934437

[29] McGuone D Crandall LG Devinsky O. Sudden unexplained death in childhood: a neuropathology review. Front Neurol. (2020) 11:582051. 10.3389/fneur.2020.582051 33178125 PMC7596260

[30] McGuone D Leitner D William C Faustin A Leelatian N Reichard R. Neuropathologic changes in sudden unexplained death in childhood. J Neuropathol Exp Neurol. (2020) 79:336–46. 10.1093/jnen/nlz136 31995186 PMC7036658

[31] Roy A Millen KJ Kapur RP. Hippocampal granule cell dispersion: a non-specific finding in pediatric patients with no history of seizures. Acta Neuropathol Commun. (2020) 8:54. 10.1186/s40478-020-00928-3 32317027 PMC7171777

[32] Leitner DF McGuone D William C Faustin A Askenazi M Snuderl M. Blinded review of hippocampal neuropathology in sudden unexplained death in childhood reveals inconsistent observations and similarities to explained paediatric deaths. Neuropathol Appl Neurobiol. (2021) 1–12. 10.1111/nan.12746. [Epub ahead of print]. 34164845 PMC8777468

[33] Azmitia EC Gannon PJ. The primate serotonergic system: a review of human and animal studies and a report on *Macaca fascicularis*. Adv Neurol. (1986) 43:407–68. 2418648

[34] Djavadian RL. Serotonin and neurogenesis in the hippocampal dentate gyrus of adult mammals. Acta Neurobiol Exp. (2004) 64:189–200. 15366252 10.55782/ane-2004-1505

[35] Kinney HC Haynes RL Armstrong DD Goldstein RD. Abnormalities of the hippocampus in sudden and unexpected death in early life. In: Duncan JR Byard RW editors. SIDS Sudden Infant and Early Childhood Death: The Past, the Present and the Future. Adelaide, SA: University of Adelaide Press (2018). p. 661–88. 30035970

[36] Toczek MT Carson RE Lang L Ma Y Spanaki MV Der MG. PET imaging of 5-HT1A receptor binding in patients with temporal lobe epilepsy. Neurology. (2003) 60:749–56. 10.1212/01.WNL.0000049930.93113.20 12629228

[37] Savic I Lindstrom P Gulyas B Halldin C Andree B Farde L. Limbic reductions of 5-HT1A receptor binding in human temporal lobe epilepsy. Neurology. (2004) 62:1343–51. 10.1212/01.WNL.0000123696.98166.AF 15111672

[38] Cerpa VJ Aylwin Mde L Beltran-Castillo S Bravo EU Llona IR Richerson GB. The alteration of neonatal raphe neurons by prenatal-perinatal nicotine. Meaning for sudden infant death syndrome. Am J Respir Cell Mol Biol. (2015) 53:489–99. 10.1165/rcmb.2014-0329OC 25695895 PMC4742896

[39] Duncan JR Garland M Myers MM Fifer WP Yang M Kinney HC. Prenatal nicotine-exposure alters fetal autonomic activity and medullary neurotransmitter receptors: implications for sudden infant death syndrome. J Appl Physiol. (2009) 107:1579–90. 10.1152/japplphysiol.91629.2008 19729586 PMC2777800

[40] Polli FS Ipsen TH Caballero-Puntiverio M Osterbog TB Aznar S Andreasen JT. Cellular and molecular changes in hippocampal glutamate signaling and alterations in learning, attention, and impulsivity following prenatal nicotine exposure. Mol Neurobiol. (2020) 57:2002–20. 10.1007/s12035-019-01854-9 31916029

[41] Zeid D Kutlu MG Gould TJ. Differential effects of nicotine exposure on the hippocampus across lifespan. Curr Neuropharmacol. (2018) 16:388–402. 10.2174/1570159X15666170714092436 28714396 PMC6018186

[42] Ostfeld BM Schwartz-Soicher O Reichman NE Teitler JO Hegyi T. Prematurity and sudden unexpected infant deaths in the United States. Pediatrics. (2017) 140:e20163334. 10.1542/peds.2016-3334 28759397

[43] Duncan JR Byard RW. Sudden infant death syndrome: an overview. In: Duncan JR Byard RW editors. SIDS Sudden Infant and Early Childhood Death: The Past, the Present and the Future. Adelaide, SA: University of Adelaide Press (2018). p. 15–50.

